# Supplementary material for: Herbicide risk assessments of non-target terrestrial plant communities: A graphical user interface for the plant community model IBC-grass
Source: PLoS One. 2020 Mar 13;15(3):e0230012. doi: 10.1371/journal.pone.0230012 (PMC7069634; doi:10.1371/journal.pone.0230012)
Supplement: S1 File — (DOCX) [file pone.0230012.s001.docx]

Appendix A: Long-term validation of IBC-grass

# Experimental long-term data

IBC-grass was parameterized according to and afterwards compared with the long-term experimental data set of the Kalø plot in Denmark (Damgaard et al., 2016; Strandberg et al., 2012). In 2001, a long-term experiment was established investigating the impact of the herbicide glyphosate and fertilizer on a local plant community. The experiment was established on a former agricultural field, which was deeply ploughed before the beginning of the experiment. In a randomized block design, plots with different levels of herbicide and fertilizer treatments were established on that field. 4 different herbicide treatments (0, 14.4, 72 and 360 g/ha) and 3 different fertilizer treatments (0, 25, 100 kg N/ha) were considered on 7x7m² plots with 10 replicates each. Plant cover was estimated on sub plots using the pin-point method.

We used cover data published in Strandberg et al. (2012) and Damgaard et al. (2016) to parameterize according to the control data and to compare the treatment data with model predictions (Table 1, Table 2).

Table 1: Cover values published in Strandberg et al. (2012) with no nitrogen input.

| Year | Application rate | Species | Cover | | |
| --- | --- | --- | --- | --- | --- |
| 2007 | 0 | *Festuca ovina* | 0.62 | 0.77 |  |
|  |  | *Elytrigia repens* | 0.05 | 0.08 |  |
|  |  | *Agrostis capillaris* | 0.18 | 0.29 |  |
|  | 14 | *Festuca ovina* | 0.55 | 0.60 |  |
|  |  | *Elytrigia repens* | 0.01 | 0.01 |  |
|  |  | *Agrostis capillaris* | 0.46 | 0.21 |  |
|  | 72 | *Festuca ovina* | 0.65 | 0.55 |  |
|  |  | *Elytrigia repens* | 0 | 0.01 |  |
|  |  | *Agrostis capillaris* | 0.44 | 0.3 |  |
|  | 360 | *Festuca ovina* | 0.7 | 0.75 |  |
|  |  | *Elytrigia repens* | 0 | 0 |  |
|  |  | *Agrostis capillaris* | 0.2 | 0.18 |  |
| 2008 | 0 | *Festuca ovina* | 0.47 | 0.53 | 0.76 |
|  |  | *Elytrigia repens* | 0.05 | 0.06 | 0.06 |
|  |  | *Agrostis capillaris* | 0.18 | 0.18 | 0.12 |
|  | 14 | *Festuca ovina* | 0.4 | 0.52 | 0.61 |
|  |  | *Elytrigia repens* | 0.05 | 0.05 | 0.02 |
|  |  | *Agrostis capillaris* | 0.17 | 0.18 | 0.21 |
|  | 72 | *Festuca ovina* | 0.7 | 0.57 | 0.45 |
|  |  | *Elytrigia repens* | 0.01 | 0.005 | 0 |
|  |  | *Agrostis capillaris* | 0.25 | 0.16 | 0.2 |
|  | 360 | *Festuca ovina* | 0.59 | 0.61 | 0.79 |
|  |  | *Elytrigia repens* | 0 | 0 | 0 |
|  |  | *Agrostis capillaris* | 0.07 | 0.02 | 0.07 |

Table 2: Cover values published in Damgaard et al. (2016) with no nitrogen input.

| Year | Application rate | Species | Cover |
| --- | --- | --- | --- |
| 2005 | 0 | no plants | 0.05 |
|  |  | *Festuca ovina* | 0.75 |
|  |  | *Tanacetum vulgare* | 0.05 |
|  |  | *Euphorbia esula* | 0.01 |
|  |  | *Agrostis gigantea* | 0.11 |
|  |  | *Hieracium pilosella* | 0.15 |
|  |  | *Elytrigia repens* | 0.05 |
|  |  | *Leucanthemum vulgare* | 0.035 |
|  |  | *Linaria vulgaris* | 0.01 |
|  | 14 | no plants | 0.04 |
|  |  | *Festuca ovina* | 0.65 |
|  |  | *Tanacetum vulgare* | 0.05 |
|  |  | *Euphorbia esula* | 0.02 |
|  |  | *Agrostis gigantea* | 0.11 |
|  |  | *Hieracium pilosella* | 0.155 |
|  |  | *Elytrigia repens* | 0.05 |
|  |  | *Leucanthemum vulgare* | 0.07 |
|  |  | *Linaria vulgaris* | 0.01 |
|  | 72 | no plants | 0.05 |
|  |  | *Festuca ovina* | 0.7 |
|  |  | *Tanacetum vulgare* | 0.06 |
|  |  | *Euphorbia esula* | 0.05 |
|  |  | *Agrostis gigantea* | 0.08 |
|  |  | *Hieracium pilosella* | 0.15 |
|  |  | *Elytrigia repens* | 0.04 |
|  |  | *Leucanthemum vulgare* | 0.07 |
|  |  | *Linaria vulgaris* | 0.01 |
|  | 360 | no plants | 0.12 |
|  |  | *Festuca ovina* | 0.85 |
|  |  | *Tanacetum vulgare* | 0.01 |
|  |  | *Euphorbia esula* | 0.03 |
|  |  | *Agrostis gigantea* | 0.005 |
|  |  | *Hieracium pilosella* | 0.02 |
|  |  | *Elytrigia repens* | 0 |
|  |  | *Leucanthemum vulgare* | 0.005 |
|  |  | *Linaria vulgaris* | 0.005 |

# Model parameterization

For the parameterization of IBC-grass, we selected plots receiving no fertilizer and no herbicide input in order to be independent from the actual comparison of herbicide effects.

## PFT classification

We classified the occurring plant species to plant functional types according to plant information obtained by Beate Strandberg (personal communication, 2017) and the data bases BiolFlor, LEDA and clopla3 (Kleyer et al., 2008; Klimešová and de Bello, 2009; Klotz et al., 2002) (Table 3).

Table 3: PFT classification

| Species | annual/  biennial/  perennial | Growth form | Maximal plant mass | Resource response | Grazing response | Clonality | **Flowering period** | **Germination period** |
| --- | --- | --- | --- | --- | --- | --- | --- | --- |
| *A. campestris* | p | Semi-rosette | Medium | Stress-tolerator | Intermediate | Long spacer, resource sharing | Late | Early and late |
| *A. capillaris* | p | Semi-rosette | Medium | Intermediate | Tolerator | Long spacer, resource sharing | Late | Early and late |
| *A. gigantea* | p | Semi-rosette | Medium | Competitor | Avoider | Long spacer, resource sharing | Late | Early and late |
| *A. millefolium* | p | Semi-rosette | Medium | Competitor | Avoider | Long spacer, resource sharing | Late | Early and late |
| *A. vulgaris* | p | Semi-rosette | Large | Competitor | Tolerator | Long spacer, resource sharing | Late | Early and late |
| *C. fontanum* | p | Erect | Small | Intermediate | Tolerator | Short spacer, resource sharing | Late | Early and late |
| *C. rotundifolia* | p | Semi-rosette | Small | Stress-tolerator | Tolerator | Long spacer, resource sharing | Late | Early and late |
| *E. esula* | p | Erect | Medium | Intermediate | Avoider | Long spacer, resource sharing | Late | Early |
| *E. repens* | p | Semi-rosette | Large | Competitor | Tolerator | Long spacer, resource sharing | Late | Early and late |
| *F. ovina* | p | Semi-rosette | Small | Stress-tolerator | Tolerator | Short spacer, resource sharing | Late | Early and late |
| *F. vulgaris* | p | Semi-rosette | Medium | Intermediate | Intermediate | Short spacer, resource sharing | Late | Early and late |
| *G. aparine* | a | Erect | Medium | Competitor | Intermediate | Aclonal | Late | Early and late |
| *G. mollugo* | p | Erect | Medium | Competitor | Intermediate | Long spacer, no resource sharing | Late | Early and late |
| *H. lanatus* | p | Semi-rosette | Medium | Competitor | Tolerator | Short spacer, resource sharing | Late | Early and late |
| *H. perforatum* | p | Erect | Medium | Intermediate | Tolerator | Short spacer, resource sharing | Late | Early and late |
| *H. pilosella* | p | Rosette | Small | Stress-tolerator | Avoider | Aclonal | Late | Early and late |
| *H. radicata* | p | Rosette | Small | Intermediate | Avoider | Short spacer, resource sharing | Late | Early and late |
| *L. corniculatus* | p | Erect | Small | Intermediate | Tolerator | Short spacer, resource sharing | Late | Early and late |
| *L. vulgare* | p | Semi-rosette | Medium | Intermediate | Intermediate | Long spacer, resource sharing | Late | Early |
| *L. vulgaris* | p | Erect | Medium | Intermediate | Tolerator | Long spacer, resource sharing | Late | Early and late |
| *O. biennis* | b | Semi-rosette | Large | Intermediate | Tolerator | Short spacer, resource sharing | Late | Early and late |
| *P. angustifolia* | p | Semi-rosette | Medium | Stress-tolerator | Avoider | Long spacer, no resource sharing | Late | Early and late |
| *R. acetosella* | p | Semi-rosette | Small | Intermediate | Avoider | Long spacer, no resource sharing | Late | Early and late |
| *S. dioca* | p | Semi-rosette | Medium | Competitor | Intermediate | Long spacer, no resource sharing | Late | Early and late |
| *S. virgaurea* | p | Semi-rosette | Medium | Intermediate | Intermediate | Short spacer, resource sharing | Late | Early and late |
| *S. vulgaris* | p | Semi-rosette | Small | Competitor | Intermediate | Short spacer, resource sharing | Late | Early and late |
| *Taraxacum* | p | Rosette | Small | Intermediate | Avoider | Short spacer, resource sharing | Late | Early and late |
| *T. vulgare* | p | Semi-rosette | Medium | Competitor | Intermediate | Long spacer, resource sharing | Late | Early and late |
| *V. thapsus* | b | Semi-rosette | Large | Intermediate | Tolerator | Aclonal | Late | Early and late |
| *V. vulgaris* | p | Semi-rosette | Medium | Intermediate | Intermediate | Short spacer, resource sharing | Late | Early and late |

## Parameter fitting

The modelled patch size was set to 50x50 cm² as the pin-point method in the experiment used a 50x50cm² grid to estimate the cover. The number of initial seed input and seed input between years was set to 5 mg seeds per PFT. As the plots were not managed by grazing or mowing, we set disturbances to a low level representing grazing by small herbivores and a low number of trampling events.

Table 4: Model parameters

| PArameter | Value | Unit |
| --- | --- | --- |
| Grid size | 50 | cm² |
| Initial seeds | 5 | mg/PFT |
| Seed input | 5 | mg/PFT |
| grazing | 0.1 | fraction grazed per year |
| Trampling | 0.1 | fraction of area trampled per year |
| week of herbicide application | 10 | week of growing season |
| Amplitude for seasonal aboveground resources | 0.3 | - |
| Belowground resource units | optimized per data set* | |
| Aboveground resource units | optimized per data set* | |
| * resource units were optimized against the sum of squared differences for each data set | | |

Next, we fitted the model parameters above- and belowground resource units so that the model reflected the control data of the experiments (without herbicide and fertilizer treatments) published in Strandberg et al. (2012) and Damgaard et al. (2016) (see Table 2).

We ran an optimization routine in R (using the optim() function with the Nelder-Mead method (Nelder and Mead, 1965)), which optimized the sum of squared differences of experimental to modelled cover values by changing the above- and belowground resource units. The mean over 30 simulation runs was compared to the empirical data. As there was no clear optimum (degeneracy was indicated), we randomly selected start values (30 times) and chose only the best parameter combinations (0.5th percentile regarding the sum of squared differences, Figure 1).


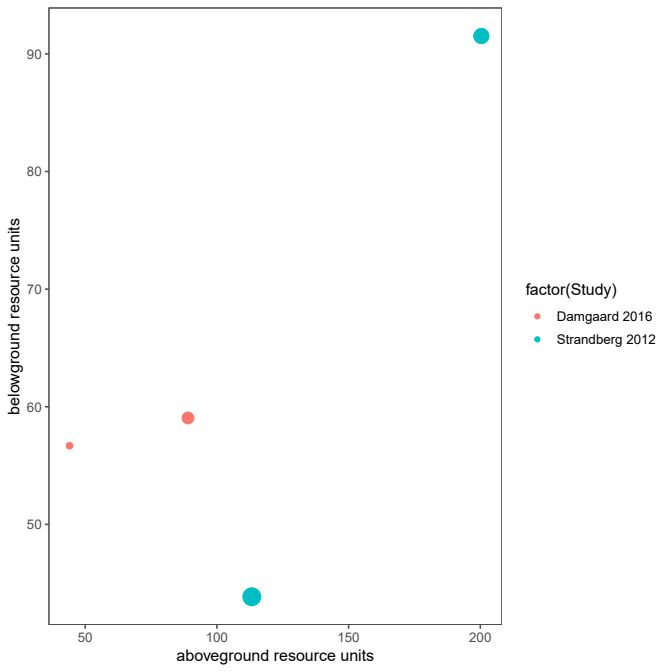


Figure 1: Optimized above- and belowground resource units with the 5% least sum of squared differences.

## Results

For both data sets, the cover of *Festuca ovina* is underestimated by the model. However, for the other species, the model prediction is close to the empirical values (Figure 2, Figure 3).

Figure 2: Comparison between modelled cover values after the optimization routine and empirically measured cover values published in Strandberg et al. (2012). Points represent the mean of the specific year and error bars the standard deviation (N=60). The dotted line represents a perfect fit of modelled vs. measured

Figure 3: Comparison between modelled cover values after the optimization routine and empirically measured cover values published in Damgaard et al. (2016). Points represent the mean of the specific year and error bars the standard deviation (N=60).

# Assessment of model performance

## Dose response curves

To apply herbicide effects, we calculated dose response curves based on effect data of a standardized greenhouse study following the OECD guideline 227 (OECD, 2006) on RoundupBio® (data is confidential). The parameters *EC50* and *b* of Equation 1 were optimized to obtain the dose responses for each test species (N=10) and measured endpoint (shoot weight, shoot length and survival). As shoot weight was more sensitive than shoot length, we used only the dose responses of shoot weight and survival.

$Effect= \frac{{AppRate}^{b}}{{EC50}^{b}+{AppRate}^{b}}$ Equation 1

As the test species did not correspond to the plant species in the experimental study, we calculated the mean and standard deviation of the estimated parameters *EC50* and *b* and assigned random dose response curves based on these values for each Monte Carlo run. As *F. ovina* showed no response to the herbicide in the experiments, we assumed that this species is not sensitive or has buffer mechanisms (see also (Damgaard et al., 2011)).

The effect on shoot weight was implemented as a reduction of biomass gain within the 10^th^ week of growing period in the model (corresponding to an herbicide application end of May/beginning of June); the effect on survival was implemented as an additional mortality probability in the 10^th^ week of the growing period in the model.

## Results

In general, the model was able to predict similar cover values compared to the empirical measurements published in Strandberg et al. (2012) and Damgaard et al. (216) for all tested application rates (Fig. 4 and Fig. 5). Especially plant species with lower cover values are well reflected in the model. The highest application rate showed the best fit of the model, especially for *F. ovina*. For *F. ovina*, the model correctly predicts the empirically measured cover values only for this high application rate.

Figure 4: Modelled cover values versus measured cover values for all application rates using the data published in Strandberg et al. (2012). The control data (application rate = 0) was used for parameterization. Points represent the mean of the model predictions of the specific year; error bars show the standard deviation. One simulation includes two combinations of above- and belowground resource units (see Model parameterization) and for each combination 30 Monte Carlo simulations with randomly assigned dose responses, except for F. ovina which was not affected.

Figure 5: Modelled cover values versus measured cover values for all application rates using the data published in Damgaard et al. (2016). The control data (application rate = 0) was used for parameterization. Points represent the mean of the model predictions of the specific year; error bars show the standard deviation. One simulation includes two combinations of above- and belowground resource units (see Model parameterization) and for each combination 30 Monte Carlo simulations with randomly assigned dose responses, except for F. ovina which was not affected.

# References

Damgaard, C., Strandberg, B., Dupont, Y., Holmstrup, M., Henning Krogh, P., 2016. The effect of glyphosate and nitrogen on plant communities and the soil fauna in terrestrial biotopes at field margins. Pestic. Res.

Damgaard, C., Strandberg, B., Mathiassen, S.K., Kudsk, P., 2011. The combined effect of nitrogen and glyphosate on the competitive growth, survival and establishment of Festuca ovina and Agrostis capillaris. Agric. Ecosyst. Environ. 142, 374–381. https://doi.org/10.1016/j.agee.2011.06.008

Kleyer, M., Bekker, R.M., Knevel, I.C., Bakker, J.P., Thompson, K., Sonnenschein, M., Poschlod, P., van Groenendael, J.M., Klimes, L., Klimesová, J., Klotz, S., Rusch, G.M., Hermy, M., Adriaens, D., Boedeltje, G., Bossuyt, B., Dannemann, A., Endels, P., Götzenberger, L., Hodgson, J.G., Jackel, A.-K., Kühn, I., Kunzmann, D., Ozinga, W.A., Römermann, C., Stadler, M., Schlegelmilch, J., Steendamm, H.J., Tackenberg, O., Wilmann, B., Cornelissen, J.H.C., Eriksson, O., Garnier, E., Peco, B., 2008. The LEDA Traitbase: A database of life-history traits of Northwest European flora. J. Ecol. 96, 1266–1274.

Klimešová, J., de Bello, F., 2009. CLO-PLA: the database of clonal and bud bank traits of Central European flora. J. Veg. Sci. 20, 511–516.

Klotz, W., Kühn, S., Durka, I., 2002. BIOLFLOR - Eine Datenbank zu biologisch-ökologischen Merkmalen der Gefäßpflanzen in Deutschland, in: Klotz, W., Kühn, S., Durka, I. (Eds.), Schriftenreihe Für Vegetationskunde 38. Bundesamt für Naturschutz, Bonn.

Nelder, J.A., Mead, R., 1965. A Simplex Method for Function Minimization. Comput. J. 7, 308–313. https://doi.org/10.1093/comjnl/7.4.308

OECD, 2006. Test No. 227: Terrestrial Plant Test: Vegetative Vigour Test. OECD Guidel. Test. Chem. 21. https://doi.org/10.1787/20745761

Strandberg, B., Mathiassen, S.K., Bruus, M., Kjaer, C., Damgaard, C., Andersen, H.V., Bossi, R., Løfstrøm, P., Larsen, S.E., Bak, J., Kudsk, P., 2012. Effects of herbicides on non-target plants: How do effects in standard plant test relate to effects in natural habitats?, Pesticide Research No 137 Danish Ministry of the Environment, EPA.
