# Supplementary material for: Herbicide risk assessments of non-target terrestrial plant communities: A graphical user interface for the plant community model IBC-grass
Source: PLoS One. 2020 Mar 13;15(3):e0230012. doi: 10.1371/journal.pone.0230012 (PMC7069634; doi:10.1371/journal.pone.0230012)
Supplement: Attachment — Submitted filename: PONE-D-19-21544_reviewer.pdf [file pone.0230012.s002.pdf]

PLOS ONE

Herbicide risk assessments of non-target terrestrial plant communities: A graphical user interface for the plant community model IBC-grass

--Manuscript Draft--

**Manuscript Number:**	PONE-D-19-21544
**Article Type:**	Research Article
**Full Title:**	Herbicide risk assessments of non-target terrestrial plant communities: A graphical user interface for the plant community model IBC-grass
**Short Title:**	Herbicide risk assessment: A GUI for plant community model IBC-grass
**Corresponding Author:**	Jette Reeg<br>University of Potsdam<br>Potsdam, GERMANY
**Keywords:**	herbicide risk assessment; non-target terrestrial plant community; community modelling; software tool; ecotoxicology
**Abstract:**	Plants located adjacent to agricultural fields are important for maintaining biodiversity in semi-natural landscapes. To avoid undesired impacts on these plants due to herbicide application on the arable fields, regulatory risk assessments are conducted prior to registration to ensure proposed uses of plant protection products do not present an unacceptable risk. The current risk assessment approach for these non-target terrestrial plants (NTTPs) examines impacts at the individual-level as a surrogate approach for protecting the plant community due to the inherent difficulties of directly assessing population or community level impacts.<br><br>However, modelling approaches are suitable higher tier tools to upscale individual-level effects to community level. IBC-grass is a sophisticated plant community model, which has already been applied in several studies. However, as it is a console application software, it was not deemed sufficiently user-friendly for risk managers and assessors to be conveniently operated without prior expertise in ecological models.<br><br>Here, we present a user-friendly and open source graphical user interface (GUI) for the application of IBC-grass in regulatory herbicide risk assessment. It facilitates the use of the plant community model for predicting long-term impacts of herbicide applications on NTTP communities. The GUI offers two options to integrate herbicide impacts: (1) dose responses based on current standard experiments (acc. to testing guidelines) and (2) based on specific effect intensities. Both options represent suitable higher tier options for future risk assessments of NTTPs as well as for research on the ecological relevance of effects.
**Order of Authors:**	Jette Reeg
	Simon Heine
	Christine Mihan
	Sean McGee
	Thomas G. Preuss
	Florian Jeltsch
**Additional Information:**	
**Question**	**Response**
**Financial Disclosure**<br><br>Enter a financial disclosure statement that describes the sources of funding for the work included in this submission. Review the submission guidelines for detailed	The project was funded by Bayer AG. Authors employed by Bayer AG worked on preparing this manuscript (see Author contributions).

Powered by Editorial Manager® and ProduXion Manager® from Aries Systems Corporation

requirements. View published research articles from *PLOS ONE* for specific examples.<br><br>This statement is required for submission and **will appear in the published article** if the submission is accepted. Please make sure it is accurate.<br><br>**Unfunded studies**<br>Enter: *The author(s) received no specific funding for this work.*<br><br>**Funded studies**<br>Enter a statement with the following details:<br>• Initials of the authors who received each award<br>• Grant numbers awarded to each author<br>• The full name of each funder<br>• URL of each funder website<br>• Did the sponsors or funders play any role in the study design, data collection and analysis, decision to publish, or preparation of the manuscript?<br>• **NO** - Include this sentence at the end of your statement: *The funders had no role in study design, data collection and analysis, decision to publish, or preparation of the manuscript.*<br>• **YES** - Specify the role(s) played.<br><br>* typeset	
**Competing Interests**<br><br>Use the instructions below to enter a competing interest statement for this submission. On behalf of all authors, disclose any competing interests that could be perceived to bias this work—acknowledging all financial support and any other relevant financial or non-financial competing interests.<br><br>This statement **will appear in the published article** if the submission is accepted. Please make sure it is accurate. View published research articles from *PLOS ONE* for specific examples.	The authors have declared that no competing interests exist.

**NO authors have competing interests**<br><br>Enter: *The authors have declared that no competing interests exist.*<br><br>**Authors with competing interests**<br><br>Enter competing interest details beginning with this statement:<br><br>*I have read the journal's policy and the authors of this manuscript have the following competing interests: [insert competing interests here]*<br><br>* typeset	
**Ethics Statement**<br><br>Enter an ethics statement for this submission. This statement is required if the study involved:<br><br>• Human participants<br>• Human specimens or tissue<br>• Vertebrate animals or cephalopods<br>• Vertebrate embryos or tissues<br>• Field research<br><br>Write "N/A" if the submission does not require an ethics statement.<br><br>General guidance is provided below. Consult the submission guidelines for detailed instructions. **Make sure that all information entered here is included in the Methods section of the manuscript.**	N/A

**Format for specific study types**<br><br>**Human Subject Research (involving human participants and/or tissue)**<br>• Give the name of the institutional review board or ethics committee that approved the study<br>• Include the approval number and/or a statement indicating approval of this research<br>• Indicate the form of consent obtained (written/oral) or the reason that consent was not obtained (e.g. the data were analyzed anonymously)<br><br>**Animal Research (involving vertebrate animals, embryos or tissues)**<br>• Provide the name of the Institutional Animal Care and Use Committee (IACUC) or other relevant ethics board that reviewed the study protocol, and indicate whether they approved this research or granted a formal waiver of ethical approval<br>• Include an approval number if one was obtained<br>• If the study involved *non-human primates*, add *additional details* about animal welfare and steps taken to ameliorate suffering<br>• If anesthesia, euthanasia, or any kind of animal sacrifice is part of the study, include briefly which substances and/or methods were applied<br><br>**Field Research**<br><br>Include the following details if this study involves the collection of plant, animal, or other materials from a natural setting:<br>• Field permit number<br>• Name of the institution or relevant body that granted permission	
**Data Availability**<br><br>Authors are required to make all data underlying the findings described fully available, without restriction, and from the time of publication. PLOS allows rare exceptions to address legal and ethical concerns. See the PLOS Data Policy and FAQ for detailed information.	No - some restrictions will apply

Powered by Editorial Manager® and ProduXion Manager® from Aries Systems Corporation

A Data Availability Statement describing where the data can be found is required at submission. Your answers to this question constitute the Data Availability Statement and **will be published in the article**, if accepted.<br><br>**Important:** Stating 'data available on request from the author' is not sufficient. If your data are only available upon request, select 'No' for the first question and explain your exceptional situation in the text box.<br><br>Do the authors confirm that all data underlying the findings described in their manuscript are fully available without restriction?	
**Describe where the data may be found in full sentences. If you are copying our sample text, replace any instances of XXX with the appropriate details.**<br><br>• If the data are **held or will be held in a public repository**, include URLs, accession numbers or DOIs. If this information will only be available after acceptance, indicate this by ticking the box below. For example: *All XXX files are available from the XXX database (accession number(s) XXX, XXX.).*<br>• If the data are all contained **within the manuscript and/or Supporting Information files**, enter the following: *All relevant data are within the manuscript and its Supporting Information files.*<br>• If neither of these applies but you are able to provide **details of access elsewhere**, with or without limitations, please do so. For example:<br><br>*Data cannot be shared publicly because of [XXX]. Data are available from the XXX Institutional Data Access / Ethics Committee (contact via XXX) for researchers who meet the criteria for access to confidential data.*<br><br>*The data underlying the results presented in the study are available from (include the name of the third party*	All software files and additional information are available at https://github.com/JetteReeg/IBCgrassGUI.<br>Additional relevant data are within the manuscript and the Supporting Information file, except for the confidential dose-response data used in the supporting information file.

Powered by Editorial Manager® and ProduXion Manager® from Aries Systems Corporation

and contact information or URL).<br>• This text is appropriate if the data are owned by a third party and authors do not have permission to share the data.<br><br>* typeset	
Additional data availability information:	

Powered by Editorial Manager® and ProduXion Manager® from Aries Systems Corporation

Manuscript
Click here to download Manuscript Reeg_final_withoutMendeley_2.docx

Title: Herbicide risk assessments of non-target terrestrial plant communities: A graphical user interface for the plant community model IBC-grass

Authors:

Jette Reeg[1*], Simon Heine[2], Christine Mihan[2], Sean McGee[3], Thomas G. Preuss[2] and Florian Jeltsch[1,4]

[1] Plant Ecology and Nature Conservation, University of Potsdam, Potsdam, Germany

[2] Bayer AG, Monheim am Rhein, Germany,

[3] Bayer CropScience, Research Triangle Park, North Carolina, USA

[4] Berlin-Brandenburg Institute of Advances Biodiversity Research, Berlin, Germany

* Corresponding author: jreeg@uni-potsdam.de

ABSTRACT

Plants located adjacent to agricultural fields are important for maintaining biodiversity in semi-natural landscapes. To avoid undesired impacts on these plants due to herbicide application on the arable fields, regulatory risk assessments are conducted prior to registration to ensure proposed uses of plant protection products do not present an unacceptable risk. The current risk assessment approach for these non-target terrestrial plants (NTTPs) examines impacts at the individual-level as a surrogate approach for protecting the plant community due to the inherent difficulties of directly assessing population or community level impacts.

However, modelling approaches are suitable higher tier tools to upscale individual-level effects to community level. IBC-grass is a sophisticated plant community model, which has already been applied in several studies. However, as it is a console application software, it was not deemed sufficiently user-friendly for risk managers and assessors to be conveniently operated without prior expertise in ecological models.

Here, we present a user-friendly and open source graphical user interface (GUI) for the application of IBC-grass in regulatory herbicide risk assessment. It facilitates the use of the plant community model for predicting long-term impacts of herbicide applications on NTTP communities. The GUI offers two options to integrate herbicide impacts: (1) dose responses based on current standard experiments (acc. to testing guidelines) and (2) based on specific effect intensities. Both options represent suitable higher tier options for future risk assessments of NTTPs as well as for research on the ecological relevance of effects.

INTRODUCTION

Agricultural land covers more than half of the terrestrial landscape in Europe. Especially in intensively managed croplands, agricultural practices may lead to undesired impacts on semi-natural landscape structures such as field boundaries or hedgerows. For example depending on wind conditions, the application of herbicides on crop fields may reach non-target areas such as field boundaries through

drift. This can lead to unintended effects on non-target plant communities. As semi-natural landscapes play an important role in maintaining biodiversity in agricultural landscapes, for instance by providing food or serving as shelter habitat [1,2], there is a need for protecting these field margins from unacceptable adverse impacts caused by agricultural practices. Therefore, before a new product is placed on the market, a risk assessment is conducted to evaluate the potential risk of applying the product according to the proposed label specifications [3].

The current risk assessment scheme for non-target terrestrial plants (NTTPs), i.e. plants not intended to be affected by the plant protection product, follows a tiered approach. The baseline risk assessment is based on standardized greenhouse experiments to test for impacts on vegetative vigour and seedling emergence at the individual plant level under different application rates (OECD testing guidelines 208 and 227 [4,5]). These studies are not designed to cover inter- and intraspecific competition between plant individuals in a plant community. In general, assessment factors are applied to account for uncertainties such as the extrapolation from greenhouse to the field or the existence of even more sensitive species. Guidance on harmonized and fully accepted higher tier studies, which could potentially overcome some of that uncertainty, is not available.

Modelling approaches are often mentioned as suitable tools for higher tier evaluations [6]. As ecological models are limited by computational resources only, they overcome the spatial and temporal limitations as well as high resource requirements of empirical field studies. Thus, a range of different environmental conditions can be tested as a full factorial design. While relevant stakeholders (e.g., risk assessors and risk managers) have expert knowledge with the ecological aspects and empirical data used in ecological modelling, they may lack experience with computational aspects of models. Therefore ecological models need to fulfil certain requirements to be considered as suitable higher tier approaches: (1) models need to be validated against empirical data, proving that the simplified model is able to reflect real world conditions [7]; (2) a sensitivity analyses of model parameters that are not based on empirical data need to show the robustness of the model; (3) a comprehensive model documentation should facilitate the communication between model

developers and users, e.g. regulators, by presenting the applicability and capabilities of the model [8,9]. These requirements are essential to establish trust in the models' capabilities but also to reveal possible limitations that come along with the simplification of the real ecological system.

The plant community model IBC-grass (Individual-Based plant Community model for GRASSlands) is a suitable approach to extrapolate individual-level effects measured in standard guideline studies [5,10] to plant populations in community context. Plant community dynamics emerge from individual plant behaviour determined by plant trait characteristics. Recent studies highlighted the capability of the model to detect herbicide induced impacts on plant communities [11], showed different sensitivities of important plant attributes [12] and validated IBC-grass against short-term [13] and long-term empirical data (see supporting information file S1 File). Model development is documented using the ODD protocol (Overview, Design concept and Details [14]). Although all requirements mentioned earlier are fulfilled, the model was, up to now, not convenient to use as it was developed as a console application, which would likely lead to hesitation to apply the model, especially for researchers not trained in modelling. Here, we present a graphical user interface (GUI) for IBC-grass to facilitate the application in herbicide risk assessments. The GUI guides the user through the parameter settings, runs simulations and directly analyses the model predictions for herbicide risk assessments. The software package comes with a manual and additional model documentation, namely a Good Modelling Practice document (GMP, [8]) and a Doxygen documentation to facilitate the understanding of the source code [15].

METHODS

The model IBC-grass

In the following we will only give a broad overview of the main processes and principles of IBC-grass. A detailed model documentation can be found in the software package (ODD and GMP documents [16]).

Trait-based approach

Plant trait characteristics are known to influence plant community dynamics [17]. Species with similar set of trait characteristics, a plant functional type (PFT), are known to respond similar to environmental conditions. Thus, the PFT approach can be used to make conclusions for several plant communities consisting of different plant species but similar PFTs. In IBC-grass plant species are classified into plant functional types (PFTs) according to selected trait characteristics (Table 1) known to be important for population dynamics. They include several trade-off or correlations, e.g. seed mass and plant mass. Three different trait data bases are used to collect the corresponding trait values and to classify plant species into the different PFTs [18–20]. Each PFT is assigned a specific identification acronym, which consists of the major trait characteristics (Table 2). The GUI gives examples for each PFT ID.

Table 1: Classification of plant species into plant functional types (PFTs)

TRAIT	VALUES	BASED ON DATEBASE TRAIT	CORRESPONDING MODEL PARAMETERS		
**Plant size**		*seed releasing height*[1]	*maximal plant mass*	*seed mass*	*seed dispersal*
	small	<=0.42	1000 mg	0.1 mg	0.6 m
	medium	0.42-0.87	2000 mg	0.3 mg	0.3 m
	tall	>0.87	5000 mg	1 mg	0.1 m
**Growth form**		*rosette attribute*[2]	*leaf mass ratio*		
	erect	erect	0.5		
	semi-rosette	semi-rosette	0.75		
	rosette	rosette	1.0		
**Resource response**		*ecological strategy*[2]	*maximal resource units*	*maximal survival under resource stress*	
	stress-tolerator	sr, cs, s	20	6	
	intermediate	csr, r	40	4	
	competitor	c, cr	60	2	
**Grazing response**		*grazing tolerance*[2]	*palatability*	*specific leaf area*	
	tolerator	4-6 (resprouter)	1.0	1.0	
	intermediate	1-3 (no adaption)	0.5	0.75	
	avoider	7-9 (defence strategies)	0.25	0.5	
**Clonal type**		*clonality* [3]	*spacer length*	*resource sharing*	
	long spacer with resource sharing	lateral spread 0.01-0.25 m/y with persistence of connection	17.5 cm	1	
	long spacer without resource sharing	lateral spread 0.01-0.25 m/y with persistence of connection	17.5 cm	0	
	short spacer with resource sharing	lateral spread < 0.01m/y with persistence of connection	2.5 cm	1	
	short spacer without resource sharing	lateral spread < 0.01m/y with persistence of connection	2.5 cm	0	
**Flowering type**		*symphenological groups*[2]	*start of seed production*	*end of seed production*	
	early	1-6	week 1	week 5	
	late	7-10	week 16	week 20	
**Germination periods**			establishment period		
	spring		weeks 1-4		
	summer		weeks 21-25		
	spring and summer		week 1-4 and 21-25		

[1] [18] [2] [19] [3] [20]

Table 2: Compilation of the specific PFT ID according to the major traits

PLANT SIZE	GROWTH FORM	RESOURCE RESPONSE TYPE	GRAZING RESPONSE TYPE	CLONAL TYPE	LIFE SPAN	FLOWERING PERIOD	GERMINATION PERIOD
Small	Erect	Competitor	Avoider	cl1 short internodes, resource sharing	perennial	early	early
Medium	Semi-rosette	Stress-tolerator	Intermediate	cl2 short internodes, no resource sharing	annual	late	late
Large	Rosette	Intermediate	Tolerator	cl3 long internodes, resource sharing			both
				cl4 long internodes, no resource sharing			

2-layer zone of influence approach

To account for competition between plant individuals, a 2-layer zone of influence approach is implemented for the above- and belowground compartments: Depending on the growth form, plant size and specific leaf area/root area, each plant individual has a specific zone of influence, a circular area in which it takes up resources (Figure 1). In overlapping zones of influences, plant individuals compete for resources; intraspecific competition being stronger than interspecific competition. Aboveground, the competition is size asymmetric: taller plants shade smaller plants and thus acquire more resources. Belowground, on the other hand, competition is size symmetric: resource distribution between plant individuals with overlapping zones of influences is independent of the root mass.

Figure 1: Graphical scheme of the IBC-grass model. Graphic is adapted from Weiß [21]

Spatial and temporal dimensions

IBS-grass is a spatially explicit model: it simulates plant community dynamics on a grid 1x1 cm² cells, representing a patch in a landscape. The size of the grid can vary between 100x100 cm² (= 1 m²) and 173x173 cm² (3 m²). The local patch is simulated as a torus, i.e. the edges are connected to each other. Temporal dynamics are simulated in weekly time steps with only the growing period of spring to autumn is considered. During the winter period, a winter dieback of shoot mass and winter mortality is simulated.

Main processes

Plant growth, reproduction and mortality. Resources acquired after competition (see above) are allocated into the roots and shoots of a plant individual and converted into biomass, considering the constant conversion rate g, the current shoot (root) mass $m_{shoot(root)}$, the trait characteristics of the shoot (root) c_{shoot} (RAR), the growth form f_{leaf}, the maximal resource utilization ru_{max} and the maximal plant mass m_{max} (see ODD protocol for further details [16]). PFTs with vegetative reproduction invest 5% of their resources into the growth of one spacer (rhizome or stolon connecting ramets of clona[illegible] plants). As soon as the spacer reaches its maximal distance, spacer growth stops and the new ramet establishes with a constant probability p_{ram} of 50%. However, establishment can only take place if the grid cell is not occupied by another plant individual; otherwise spacer growth will continue within a radius of two grid cells. Once the ramet established, a new spacer is developed.

During the flowering period of the PFT (4 weeks either at the beginning of the growing season in spring or 4 weeks at the end of the growing season in late summer) plant individuals invest 5% of their acquired resources in the production of seeds [22]. In the consecutive week of this period, seeds are dispersed onto the grid. The direction is drawn from a uniform distribution and the dispersal distance depends on the PFT specific seed characteristics and is drawn from a log-normal distribution [23]. During one or two seed establishment periods, depending on the PFT specific germination type (early, late or both), seeds germinate with a probability of 50% and establish on bare ground (i.e. on cells which are not covered by any other individual). The chance of successful establishment depends on the seed mass with heavier seeds assumed to be more competitive.

On a weekly basis, plant individuals can die due to two processes: resource stress and a random, density-dependent background mortality. The mortality probability increases the longer a plant individual experiences resource stress. Thereby, the duration a plant individual can cope with resource stress depends on PFT specific trait characteristics. The random background mortality is set to 0.7% (according to an annual mortality of 20% [22]). To account for density-dependent impacts, for instance

pests, the background mortality increases exponentially with increasing population size of the specific PFT. Winter dieback of aboveground biomass is simulated as 50% biomass reduction between simulated years. Plants with a resulting biomass below 10 mg are considered as dead. Additionally, seeds suffer from winter mortality with a probability of 50% and can die due to age (maximal age = 1 as there is no long-term seed back simulated).

Disturbances. IBC-grass accounts for three disturbances in grassland systems: grazing, trampling and cutting. Grazing is simulated as the removal of biomass depending on the palatability of the PFTs. Trampling is integrated as the complete removal of aboveground biomass in 10 cm² patches, which reflect footprints. Cutting is the removal of aboveground biomass up to a specific cutting height. The height is set to 500 mg dry mass left per plant individual of PFTs with a rosette growth form (mass_left = 500 mg dry mass * f_{leaf}^2), leading to 125 mg dry mass left for erect growing plants and 281,25 mg dry mass left for semi-rosette PFTs.

Herbicide induced impacts

Herbicides can impair different plant attributes: growth, survival and reproduction. In IBC-grass six different attributes can be influences by herbicide application:

(1) Plant biomass gain: The biomass gain of a plant is reduced by a specific effect intensity/rate.
(2) Plant mortality: Based on a specific effect intensity/rate, plant suffer from an additional mortality probability.
(3) Seed number: The number of produced seeds is reduced by a specific effect intensity/rate.
(4) Seed sterility: Based on a specific effect intensity/rate, seeds suffer from an additional mortality probability.
(5) Seed establishment: The establishment probability of the seedling is reduced by a specific effect intensity/rate.
(6) Seedling biomass: Seedling biomass is reduced by a specific effect intensity/rate.

The specific effect intensity can be based either on dose responses calculated from greenhouse experiments or be specified by the user (see Section The graphical user interface for more details).

The graphical user interface

The graphical user interface (GUI) facilitates the application of IBC-grass in herbicide risk assessments. The GUI guides the user through the environmental settings and herbicide settings of IBC-grass, and analyses the results of a set of simulations (see Table 3 for an overview of the model parameters addressed in the GUI). It is an open access software hosted on GitHub [24]. The GUI itself is written in R using the R package RGtk2 [25]. The package includes a folder with the C++ source code files of the plant community model IBC-grass and a folder including the model documentation (ODD protocol [14] and GMP document [8]) and the detailed manual for the GUI. Here, we will only give a summary of the GUI. For detailed information, please have a look at the manual [16].

Table 3: Model parameters addressed in the GUI

IBC-GRASS PARAMETER	EXPLANATION
**IBCcommunity**	PFT community
**IBCgridsize**	Number of grid cells
**IBCabampl**	Amplitude for aboveground resource seasonality
**IBCabres**	Aboveground resource units
**IBCbelres**	Belowground resource units
**IBCSeedInput**	Number of seeds per PFT added at the beginning of each year
**IBCcut**	Number of cutting events per year
**IBCgraz**	Amount of area grazed during one year
**IBCtramp**	Amount of area trampled during one year
**IBCInit**	Number of initial years
**IBCDuration**	Number of year with simulated herbicide application
**IBCRecovery**	Number of years following the herbicide application period
**IBCweekstart**	Calendar week of herbicide application
**IBCherbeffect**	If 'txt-file': herbicide effects are based on a txt-file (predict potential effects)<br>If 'dose-response': herbicide effects are based on dose-response data
**IBCApprateScenarios**	Annual application rates for each scenario (if herbicide effects are based on dose-response data)
**BiomassEff**	Is plant biomass affected?
**EstablishmentEff**	Is seed establishment affected?
**SeedlingBiomassEff**	Is seedling biomass affected?
**SeedNumberEff**	Is seed number affected?
**SeedSterilityEff**	Is seed sterility affected?
**SurvivalEff**	Is plant survival affected?
**IBCrepetition**	Number of repetitions

Requirements

To run the GUI the following software needs to be installed on the local machine:

- R [26]
- G++ compiler (e.g., MingGW compiler [27])
- In some cases you might need to install GTK+ 3 [28] on your own. However, the GUI will at least try to install it on windows systems.

The R and the compiler software need to be set as environmental variable.

The GUI was tested under Windows (7 and 10) and Linux (Ubuntu 16.04). Under Mac, there might be a problem with installing GTK+ 3, thus a support for Mac iOS is not ensured.

Regional PFT pool

The GUI includes three regional PFT communities: a common field edge community with high resource input, medium trampling events and one mowing per year; *Calthion* as a nutrient poor grassland with low disturbances by grazing and trampling and one mowing event per year; and *Arrhenatheretalia* as a nutrient rich grassland with high disturbances by grazing and trampling and three mowing events per year. All three communities were used in an early study by Reeg et al. [11].

In addition to the predefined communities, the user has the possibility to create new plant communities either by selecting plant species from one of the three communities mentioned above, or by classifying new plant species into plant functional types (PFTs, Table 1).

Environmental settings

For the predefined plant communities the GUI suggests the environmental settings that were applied in Reeg et al. [11]. Nevertheless, the setting can always be adjusted.

Resource settings. Resources are given in resource units (ru) per cm^2, not defining a specific resource. 40 ru/cm^2 represent low resource levels, 100 ru/cm^2 represent high resource levels. Resources of the above- and belowground compartment are differentiated. For the aboveground resources a seasonality effect can be included: The aboveground resources can follow a sine curve. The selected amplitude determines the height of the sine curve. This seasonality can be based on light intensity data.

Disturbance settings. Three different disturbances are distinguished: grazing, trampling and cutting. Trampling removes the aboveground biomass in x% cells of the grid over the year, with one footprint being reflected as a 10 cm^2 patch. Grazing only removes a certain percentage of the aboveground shoot mass of plant individuals depending on the palatability of the PFT. Cutting events can occur 1, 2, 3 times a year or never. In one cutting event, the aboveground biomass in the whole grid is removed to a specific cutting height. The shoot mass left after a cutting event depends on the growth form specific to translate shoot mass removal to a cutting height (see ODD protocol for further details [16]).

Herbicide settings

The user has the option to vary the number of years for the initial phase (without herbicide application to stabilize the plant community), herbicide application phase including the timing of the application and recovery phase (without herbicide application). To distinguish herbicide-induced impacts from ecological impacts induced by the model initialization it is important to start herbicide application after the community dynamics stabilized (~25-50 years). Otherwise herbicide impacts might be shaded by high variation between simulation runs.

Six different plant attributes can be selected to be affected by the herbicide: shoot mass, seedling shoot mass, survival, establishment, seed sterility and seed number.

Ideally, herbicide effects are based on dose responses following the current OECD guidance documents 208 and 227 [5,10]. The user is asked to transfer the results of the experiments (specifically the number of test species and for each selected attribute the test rate and measured data per test species). The

GUI will then calculate dose responses by optimizing the parameters *EC50* and slope *b* of the Equation 1 to the empirical data using the Nelder-Mead method [29].

$$Effect\ (Application\ rate) = \frac{Application\ rate^{b}}{ER50^{b} + Application\ rate^{b}} \qquad \text{Equation 1}$$

However, if the user has no access to dose response data, the GUI offers an alternative approach, which was also used in Reeg et al. [12]: The user can specify effect intensities for each plant attribute in each year of simulated herbicide application. In this way, the user hast the possibility to analyse whether a certain individual-level effect intensity has a significant impact on population- and/or community-level.

PFT sensitivity

If the herbicide effects are based on dose-response data, the user needs to assign dose-responses to the PFTs in the plant community model. Either the calculated dose responses can be assigned directly, for instance if a plant species of a certain PFT was tested. However, the sensitivity of many non-crop plant species is still unknown, but Christl et al. [30] showed that the sensitivities of crop vs. weed species are similar. Therefore, we suggest assigning random dose responses within the variation of the calculated ones by calculating the mean and standard deviation of both the estimated *EC50* and slope *b* values and then choosing randomly from a uniform distribution within the interval of mean +- standard deviation for each single simulation (Figure 2). In addition to direct and random assignment of dose-responses, a PFT can also be assigned as being not affected at all, for example if a selective herbicide is simulated.

Figure 2: Example of calculated dose responses based on empirical data of 5 plant species. Red line represent the mean dose response (with mean of the estimated *EC50* and slope *b*), orange lines represent 100 random dose responses based on the mean and standard deviation of the estimated *EC50* and slope *b*.

On the other hand, if the user specified only certain effect intensities, the sensitivity of the different PFTs can be assigned as random (0-1), not affected (0), low (0.1-0.35), medium (0.35-0.65), high (0.65-1) or full (1). The effect intensity is multiplied by the random number out of the certain interval. For

example, if the effect intensity for plant survival was set to 0.5 and the sensitivity of a PFT to low, a random number is drawn between 0.1 and 0.35, e.g. 0.2. The resulting PFT specific effect is 0.1, which means that plant individuals of this PFT have an herbicide-induced mortality probability of 10%.

Simulation settings

In a last step, the user specifies the number of repetitions, the simulated plot size, the degree of isolation (as external seed input) and, if herbicide effects are based on dose responses, the number of different herbicide scenarios. The number of repetitions is the number of simulations that have the same model settings in the environmental and herbicide parameters (Monte-Carlo runs).

Before the simulations are started, the user need to specify the annual application rates per scenario. Especially the number of repetitions, the plot size and the number of simulated application rates have a high impact on the running time. To accelerate the running time, the GUI is parallelizing simulations using all cores but two, i.e. if a local machine has four cores, the GUI will use two of them to parallelize the IBC-grass Monte-Carlo simulations.

Analyses

Raw output data of one simulation run include responses on population- and community-level in weekly time steps: PFT population size, shoot mass and cover on population-level and number of PFTs, number of individuals, aboveground biomass and four different diversity indices (Evenness, Shannon, Simpson and inverse Simpson) on community-level. The GUI will further analyse these data, however, the user can keep the raw data for individual analyses. Note that storage footprint can be very high.

For each endpoint, the mean of the control simulation is calculated per weekly time step. Next, for all simulation runs (control and treatments), the value per time step is divided by the mean of the control to calculate effects relative to the control mean (i.e. 0.7 represents 30% reduction compared to the mean of the control). These data are saved in the files '*resultsPFT.txt*' for population-level endpoints and '*resultsGRD.txt'* for community-level endpoints. Further, the effects are averaged (mean (mean effect), 2.5th percentile (maximal effect) and 97.5th percentile (minimal effect)) over all simulations. On

the one hand per time step, but also per year. Results are saved as ‘*effect.timestep.PFT.txt*’ and ‘*effect.year.PFT.txt*’ for population level endpoints and ‘*effect.timestep.GRD.txt*’ and ‘*effect.year.GRD.txt*’ for community level endpoints. Based on the weekly analyses (‘*effect.timestep.**’), the number of weeks in which the (mean, minimum, maximum) effect is within a certain interval are summed up. For this, we used effect intervals of <10%, 10-20%, 20-30%, 30-40% and >50%. The results are saved for each endpoint separately as *‘*_PFT.txt*’ for population level endpoints and *‘*_GRD.txt’* for community level endpoints. Note that positive effects will be included in the interval <10%.

EXAMPLES

The IBC-grass GUI package includes two examples: one for herbicide effects based on dose response data and one for herbicide effects based on specific effect intensities. Here we present selected examples of potential output of the GUI only for the first example. However, as both examples are included in the GUI package, the user can load both projects to look at the full set of results.

Effects based on dose-responses

Project settings

This example simulated the impact of a potential herbicide for PFT populations of a field edge community. The herbicide effect was simulated for 10 years after an initialization phase of 35 years. The herbicide effects were based on dose-responses for impacts on biomass and mortality of 5 test species. Dose responses were randomly sampled for each PFT in each Monte Carlo simulation. The simulation settings are summarized in Table 4.

Table 4: Summary of simulation settings for the two examples: herbicide effects based on dose response data and based on specific effect intensities. Only results for the examples of the integration of dose-response data is shown later.

IBC-GRASS PARAMETER	DOSE RESPONSE	SPECIFIC EFFECT INTENSITIES
**IBCcommunity**	Fieldedge.txt	Fieldedge.txt
**IBCgridsize**	173	173
**IBCabampl**	0.0	0.0
**IBCabres**	100	100
**IBCbelres**	90	90
**IBCSeedInput**	10	10
**IBCcut**	1	1
**IBCgraz**	0.001	0.001
**IBCtramp**	0.1	0.1
**IBCInit**	35	25
**IBCDuration**	10	10
**IBCweekstart**	11	11
**IBCRecovery**	5	5
**IBCherbeffect**	Dose-response	Txt-file
**IBCAppratеScenarios**	1.1, 3.3 g a.i./ha (no annual variation)	-
**BIOMASSEFF**	TRUE	TRUE
**ESTABLISHMENTEFF**	FALSE	TRUE
**SEEDLINGBIOMASSEFF**	FALSE	FALSE
**SEEDNUMBEREFF**	FALSE	FALSE
**SeedSterilityEff**	FALSE	FALSE
**SurvivalEff**	TRUE	TRUE
**IBCrepetition**	30	30

Results

Community-level. During the first year of herbicide application, the number of individuals is decreasing and exceeding the normal range of fluctuations (Figure 3). The effects are increasing with higher application rates. The pattern is similar for all except of one diversity index: The herbicide scenario with the lower application rate (1.1 g a.i./ha) showed no significant impact on the Evenness (i.e. the mean effect is not exceeding the range of the control simulations) (Figure 4).

Figure 3: Effect on number of plant individuals during the first year of simulated herbicide application. The theoretical herbicide had an impact on biomass and mortality. PFTs had random dose response-curves. Grey ribbons show the fluctuations within control simulations, the black line shows the mean for 1.1 g a.i./ha application rate and the orange line the mean for 3.3 g a.i./ha application rate.

Figure 4: Effect on different diversity indices during the first year of simulated herbicide application. The theoretical herbicide had an impact on biomass and mortality. PFTs had random dose response curves. Grey ribbons show the

fluctuations within control simulations, the black lines show the mean for an application rate of 1.1 g a.i./ha (herbicide scenario 1) and the orange line the mean for an application rate of 3.3 g a.i./ha (herbicide scenario 2).

Over the long-term, all diversity indices showed significant impacts (i.e. the mean effects that are outside of the control fluctuations) in most years for the second herbicide scenario with an application rate of 3.3 g a.i./ha (Figure 5). However, all indices are able to recover within the 5 years of simulated recovery period. For a lower application rate of 1.1 g a.i./ha (herbicide scenario 1), the impact is considerably lower with only a few years of significant effects.

Figure 5: Long-term impacts on the diversity indices over the simulated period. Herbicide application started in year 36 and ended in year 45. The theoretical herbicide had an impact on biomass and mortality. PFTs had random dose response curves. Grey ribbons show the fluctuations within control simulations, the black lines show the mean of the 1.1 g a.i./ha application rate (herbicide scenario 1), the orange line the mean of the 3.3 g a.i./ha application rate (herbicide scenario 2).

During the first year of simulated herbicide application, the mean effect on the number of plant individuals exceeded 20% in 3 out of the 30 week growing period for the lower application rate (1.1 g a.i./ha, herbicide scenario 1) and the threshold of 50% in 5 out of 30 weeks growing period for the higher application rate (3.3 g a.i./ha, herbicide scenario 2) (Table 5). The inverse Simpson index had a negative mean effect of 20-30% in 5 of 30 weeks growing period for the herbicide scenario 1 (1.1 g a.i./ha) and exceeded the threshold of 50% in 5 of 30 weeks growing period for herbicide scenario 2 (3.3 g a.i./ha).

Table 5: Number of weeks in which the mean (minimal and maximal) negative effect on the number of plant individuals and the inverse Simpson index is within a certain effect class. As IBC-grass simulates only 30 weeks of growing period, the maximal number of weeks is 30. The simulated herbicide application started in year 36.

	YEAR	APPLICATION RATE	<10%	10-20%	20-30%	30-40%	40-50%	>50%
NUMBER OF PLANT INDIVIDUALS	35	0	30 (0 1)	0 (0 22)	0 (0 6)	0 (0 1)	0 (0 0)	0 (0 0)
		1.1	30 (0 4)	0 (0 19)	0 (0 6)	0 (0 1)	0 (0 0)	0 (0 0)
		3.3	30 (0 0)	0 (0 22)	0 (0 5)	0 (0 3)	0 (0 0)	0 (0 0)
	36	0	30 (0 1)	0 (0 3)	0 (0 23)	0 (0 3)	0 (0 0)	0 (0 0)
		1.1	25 (4 0)	2 (1 14)	3 (0 10)	0 (0 2)	0 (0 4)	0 (0 0)
		3.3	20 (4 9)	3 (1 11)	2 (0 2)	0 (0 3)	0 (0 0)	5 (5 5)
INVERSE SIMPSON INDEX	35	0	30 (0 0)	0 (0 7)	0 (0 23)	0 (0 0)	0 (0 0)	0 (0 0)
		1.1	30 (0 0)	0 (0 5)	0 (0 5)	0 (0 0)	0 (0 0)	0 (0 0)
		3.3	30 (0 23)	0 (0 6)	0 (0 1)	0 (0 0)	0 (0 0)	0 (0 0)
	36	0	30 (0 0)	0 (0 30)	0 (0 0)	0 (0 0)	0 (0 0)	0 (0 0)
		1.1	25 (5 0)	0 (3 0)	5 (0 25)	0 (0 5)	0 (0 0)	0 (0 0)
		3.3	21 (4 0)	3 (0 0)	1 (0 21)	0 (3 0)	0 (2 4)	5 (0 5)

Population-level. The impact on population size and shoot mass is similar. Therefore, we only show the impact on population size for selected PFTs (Figure 6). Only for the simulated herbicide application rate of 3.3 g a.i./ha (herbicide scenario 2), some PFTs showed long-term decreases in population sizes (SEIIcl1peb, SECTpeb). For these PFTs, the mean effect is falling below the control range in some (SECTpeb) or all (SEIIcl1peb) years. In contrast, the PFT SEITcl1plb shows a slight increase in the mean population size, however it is only exceeding the control range in a few years. In contrast, the unstable PFT MECTplb, as indicated by a high variation within the control simulations, shows a strong increase in mean population size, even exceeding control variation. During the recovery period, starting in year 45, the population sizes of the PFTs SECTpeb and SEITcl1plb are falling back into the control variation. However, the PFTs MECTplb and SEIIcl1peb, are not able to recover within 5 years. All PFTs show no significant de- or increase in population sizes for the lower application rate (1.1 g a.i./ha, herbicide scenario 1).

Figure 6: Long-term impacts on the population sizes of selected PFTs over the simulated period. Herbicide application starts in year 36 and ended in year 45. The theoretical herbicide had an impact on biomass and mortality. PFTs had random dose response curves. Grey ribbons show the fluctuations within control simulations, the black lines show the mean of the 1.1

g a.i./ha application rate (herbicide scenario 1), the orange line the mean of the 3.3 g a.i./ha application rate (herbicide 3scenario 2).

Table 6 summarizes the negative effect extends for the selected PFTs during the first year before herbicide application (year 35) and the first year of herbicide application (year 36). All PFTs show short-term impacts on the mean population size. For the low application rate of 1.1 g a.i./ha (herbicide scenario 1), only the PFT MECTplb shows an effect >30% for 5 weeks, the other three selected PFTs only show effects <30% for not more than 5 weeks per growing season. Under the higher application rate of 3.3 g a.i./ha (herbicide scenario 2), all selected PFTs show strong short-term impacts of >50% in 5-9 weeks. However, as Figure 6 shows, the PFTs MECTplb and SEITcl1plb are able to recover from both impacts, caused by the low as well as the high rate, within one growing season. The PFT MECTplb increased in population size during the herbicide application rate and was able to keep at this higher level of population size also during the recovery period.

Table 6: Number of weeks in which the mean (minimal and maximal) negative effect on population size is within a certain effect class for two different PFTs. As IBC-grass simulates only 30 weeks of growing period, the maximal number of weeks is 30. Simulated herbicide application started in year 36.

PFT	YEAR	APPLICATION RATE	<10%	10-20%	20-30%	30-40%	40-50%	>50%
**MECTplb**	35	0	30 (0 0)	0 (0 0)	0 (0 0)	0 (0 0)	0 (0 0)	0 (0 30)
		1.1	30 (0 0)	0 (0 0)	0 (0 0)	0 (0 0)	0 (0 0)	0 (0 30)
		3.3	30 (0 0)	0 (0 0)	0 (0 0)	0 (0 0)	0 (0 0)	0 (0 30)
	36	0	30 (0 0)	0 (0 0)	0 (0 0)	0 (0 0)	0 (0 0)	0 (0 30)
		1.1	18 (0 0)	7 (0 0)	0 (0 0)	5 (0 0)	0 (0 0)	0 (0 30)
		3.3	24 (0 0)	0 (0 0)	1 (0 0)	0 (0 0)	0 (0 0)	5 (5 30)
**SECTpeb**	35	0	30 (0 0)	0 (0 0)	0 (0 14)	0 (0 10)	0 (0 5)	0 (0 1)
		1.1	30 (0 0)	0 (0 15)	0 (0 9)	0 (0 5)	0 (0 1)	0 (0 0)
		3.3	30 (0 0)	0 (0 10)	0 (0 10)	0 (0 6)	0 (0 4)	0 (0 0)
	36	0	30 (0 0)	0 (0 0)	0 (0 0)	0 (0 10)	0 (0 9)	0 (0 11)
		1.1	25 (0 0)	0 (0 0)	5 (0 0)	0 (0 1)	0 (0 18)	0 (0 11)
		3.3	23 (0 0)	2 (0 1)	0 (0 0)	0 (0 0)	0 (0 0)	5 (5 9)
**SEIlcl1peb**	35	0	30 (0 0)	0 (0 0)	0 (0 0)	0 (0 0)	0 (0 22)	0 (0 8)
		1.1	30 (0 0)	0 (0 0)	0 (0 7)	0 (0 14)	0 (0 8)	0 (0 1)
		3.3	29 (0 0)	1 (0 0)	0 (0 5)	0 (0 14)	0 (0 6)	0 (0 5)
	36	0	30 (0 0)	0 (0 0)	0 (0 0)	0 (0 4)	0 (0 6)	0 (0 20)
		1.1	28 (0 0)	2 (0 0)	0 (0 0)	0 (0 2)	0 (0 16)	0 (0 12)
		3.3	20 (0 0)	1 (0 0)	0 (0 1)	0 (0 2)	0 (0 5)	9 (0 22)
**SEITcl1plb**	35	0	30 (0 0)	0 (0 0)	0 (0 3)	0 (0 13)	0 (0 14)	0 (0 0)
		1.1	30 (0 0)	0 (0 0)	0 (0 0)	0 (0 3)	0 (0 20)	0 (0 7)
		3.3	30 (0 0)	0 (0 0)	0 (0 7)	0 (0 19)	0 (0 4)	0 (0 0)
	36	0	30 (0 0)	0 (0 0)	0 (0 14)	0 (0 15)	0 (0 1)	0 (0 0)
		1.1	25 (0 0)	4 (0 0)	1 (0 0)	0 (0 3)	0 (0 13)	0 (0 14)
		3.3	24 (0 2)	0 (0 0)	1 (0 0)	0 (0 0)	0 (4 13)	5 (1 13)

CONCLUSION

We presented a graphical user interface (GUI) of the plant community model IBC-grass to provide a user-friendly software tool, which simulates herbicide induced impacts on local non-target terrestrial plant communities. The GUI guides the user through the model parameter settings, analyses simulations and finally provides the user with a graphical output.

The plant community model, IBC-grass is not only well documented using the ODD protocol and the GMP document, but it was also validated against short-term [13] and long-term data sets (see

supporting information file S1 file). A thorough sensitivity analyses was conducted in Reeg et al. [12] investigating the sensitivity of endpoints for plant community dynamics. Overall, these studies reflect the models' capability to predict herbicide impacts on plant populations in community context based on individual-level effects.

Implications for herbicide risk assessments

Current risk assessments are based on standardized greenhouse experiments on individual-level according to commonly used guidelines (e.g. OECD 208 and 227 [5,10], OCSPP 850.4100, 850.4150, 850.4230 [31–33]). There are inherent uncertainties to this approach that cannot be covered by the currently available test systems, for instance the transfer of effects to field conditions or the influence of community dynamics. In the current approach, assessment factors shall cover for these uncertainties. In a recent scientific opinion, EFSA emphasized to use modelling studies to account for the uncertainty regarding the extrapolation to community-level [6]. Given the clear documentation, the sensitivity analyses and the two validation studies, IBC-grass represents a suitable modelling tool to investigate community-level impacts of herbicide applications. In combination with the GUI, this software is a powerful tool for higher tier herbicide risk assessments of non-target terrestrial plants by extrapolating individual-level effects to plant populations in community context.

Hosted as a GitHub repository, the software package is not only open access, but also open source (incl. the IBC-grass model source code). In this way, it is assured that it can be constantly reviewed and, consequently, improved and extended by the scientific community.

ACKNOWLEDGMENTS

We would like to thank Bayer AG for funding this project.

REFERENCES

1. Buckelew LD, Pedigo LP, Mero HM, Owen MDK, Tylka GL. Effects of Weed Management Systems on Canopy Insects in Herbicide-Resistant Soybeans. J Econ Entomol. 2000;93(5):1437–43.

Available from: https://academic.oup.com/jee/article-lookup/doi/10.1603/0022-0493-93.5.1437

2. Moreby S., Southway S. Influence of autumn applied herbicides on summer and autumn food available to birds in winter wheat fields in southern England. Agric Ecosyst Environ. 1999;72(3):285–97. Available from: https://www.sciencedirect.com/science/article/pii/S0167880999000079

3. EC (European Commission). Regulation (EC) No 1107/2009 of the European Parliament and the council of 21 October 2009 concerning the placing of plant protection products on the market and repealing Council Directives 79/117/EEC and 91/414/EEC. OJ L. 2009;309:1.

4. OECD. Test No. 208: Terrestrial Plant Test: Seedling Emergence and Seedling Growth Test. In: OECD Guidelines for the Testing of Chemicals, Section 2. Paris: OECD Publishing; 2006. Available from: http://www.oecd-ilibrary.org/environment/test-no-208-terrestrial-plant-test-seedling-emergence-and-seedling-growth-test_9789264070066-en

5. OECD. Test No. 227: Terrestrial Plant Test: Vegetative Vigour Test. In: OECD Guidelines for the Testing of Chemicals, Section 2. Paris: OECD Publishing; 2006. Available from: http://www.oecd-ilibrary.org/environment/test-no-227-terrestrial-plant-test-vegetative-vigour-test_9789264067295-en

6. EFSA PPR Panel (EFSA Panel on Plant Protection Products and their Residuals). Scientific Opinion addressing the state of the science on risk assessment of plant protection products for non-target terrestrial plants. EFSA J. 2014;12(7):163 pp. Available from: http://www.efsa.europa.eu/sites/default/files/scientific_output/files/main_documents/3800.pdf

7. Grimm V, Martin BT. Mechanistic effect modeling for ecological risk assessment: Where to go from here? Integr Environ Assess Manag. 2013;9(3):e58–63. Available from: http://doi.wiley.com/10.1002/ieam.1423

8. EFSA PPR Panel (EFSA Panel on Plant Protection Products and their Residues). Scientific Opinion on good modelling practice in the context of mechanistic effect models for risk assessment of plant protection products. EFSA J. 2014;12(3):3589–91. Available from: https://efsa.onlinelibrary.wiley.com/doi/pdf/10.2903/j.efsa.2014.3589

9. Grimm V, Augusiak J, Focks A, Frank BM, Gabsi F, Johnston ASA, et al. Towards better modelling and decision support: Documenting model development, testing, and analysis using TRACE. Ecol Modell. 2014;280:129–39. Available from: http://dx.doi.org/10.1016/j.ecolmodel.2014.01.018

10. OECD. Test guideline 208: Terrestrial plant test - Seedling emergence and seedling growth test. OECD Guidel Test Chem. 2006;227:1–21. Available from: http://www.oecd-ilibrary.org/environment/test-no-208-terrestrial-plant-test-seedling-emergence-and-seedling-growth-test_9789264070066-en

11. Reeg J, Schad T, Preuss TG, Solga A, K??rner K, Mihan C, et al. Modelling direct and indirect effects of herbicides on non-target grassland communities. Ecol Modell. 2017;348:44–55. Available from: http://dx.doi.org/10.1016/j.ecolmodel.2017.01.010

12. Reeg J, Heine S, Mihan C, Preuss TG, McGee S, Jeltsch F. Potential impact of effects on reproductive attributes induced by herbicides on a plant community. Environ Toxicol Chem. 2018;37(6):1707–22.

13. Reeg J, Heine S, Mihan C, McGee S, Preuss T, Jeltsch F. Simulation of herbicide impacts on a plant community : Comparing model predictions of the plant community model IBC-grass to empirical data. Environ Sci Eur. 2018;30(44). Available from: https://doi.org/10.1186/s12302-018-0174-9

14. Grimm V, Berger U, DeAngelis DL, Polhill JG, Giske J, Railsback SF. The ODD protocol: A review and first update. Ecol Modell. 2010;221(23):2760–8. Available from: http://dx.doi.org/10.1016/j.ecolmodel.2010.08.019

15. van Heesch D. Doxygen. [cited 2019 Apr 1]. Available from: http://www.doxygen.nl/index.html

16. GitHub. IBCgrassGUI GitHub repository. 2019 [cited 2019 May 9]. Available from: https://github.com/JetteReeg/IBCgrassGUI

17. Gross N, Bagousse-Pinguet Y Le, Liancourt P, Berdugo M, Gotelli NJ, Maestre FT. Functional trait diversity maximizes ecosystem multifunctionality. Nat Ecol Evol. 2017;1(5):0132. Available from: http://www.nature.com/articles/s41559-017-0132

18. Kleyer M, Bekker RM, Knevel IC, Bakker JP, Thompson K, Sonnenschein M, et al. The LEDA Traitbase: A database of life-history traits of Northwest European flora. J Ecol. 2008;96:1266–74.

19. Klotz W, Kühn S, Durka I. BIOLFLOR - Eine Datenbank zu biologisch-ökologischen Merkmalen der Gefäßpflanzen in Deutschland. In: Klotz W, Kühn S, Durka I, editors. Schriftenreihe für Vegetationskunde 38. Bonn: Bundesamt für Naturschutz; 2002.

20. Klimešová J, de Bello F. CLO-PLA: the database of clonal and bud bank traits of Central European flora. J Veg Sci. 2009;20:511–6.

21. Weiß L. Understanding the emergence and maintenance of biodiversity in grasslands: linking individual plant responses to community patterns. University of Potsdam, Germany; 2017.

22. Schippers P, van Groenendael JM, Vleeshouwers LM, Hunt R. Herbaceous plant strategies in disturbed habitats. Oikos. 2001;95(2):198–210.

23. Stoyan D, Wagner S. Estimating the fruit dispersion of anemochorous forest trees. Ecol Modell. 2001;145(1):35–47. Available from: http://www.sciencedirect.com/science/article/pii/S0304380001003854

24. GitHub. GitHub. [cited 2019 Apr 1]. Available from: https://github.com/

25. Lawrence M, Lang D. RGtk2: A graphical user interface toolkit for R. J Stat Softw. 2010;37(8):1–

52. Available from: http://core.kmi.open.ac.uk/download/pdf/6340243.pdf

26. R Development Core Team. R: A language and environment for statistical computing. Vienna, Austria: R Foundation for Statistical Computing; 2008. Available from: http://www.r-project.org

27. MinGW. MinGW. [cited 2019 Apr 1]. Available from: http://www.mingw.org/

28. GTK+ 3. GTK+ 3 library version 3.24.7. [cited 2019 Apr 1]. Available from: https://developer.gnome.org/gtk3/3.24/

29. Nelder JA, Mead R. A Simplex Method for Function Minimization. Comput J. 1965;7(4):308–13. Available from: https://academic.oup.com/comjnl/article-lookup/doi/10.1093/comjnl/7.4.308

30. Christl H, Morilla J, Hoen T, Zumkier U. Comparative assessment of the intrinsic sensitivity of crop species and wild plant species to plant protection products and their active substances, and potential implications for the risk assessment – A literature review. Integr Environ Assess Manag. 2018;15(2):ieam.4115. Available from: https://onlinelibrary.wiley.com/doi/abs/10.1002/ieam.4115

31. EPA (US Environmental Protection Agency). OCSPP 850.4100: Seedling Emergence and Seedling Growth. In: Office of Chemical Safety and Pollution, editor. Ecological effects test guidelines. Washington, DC: US Environmental Protection Agency; 2012.

32. EPA (US Environmental Protection Agency). OCSPP 850.4230 Early Seedling Growth Toxicity Test. In: Prevention O of CS and P, editor. Ecological effects test guidelines. Washington, DC: US Environmental Protection Agency; 2012.

33. EPA (US Environmental Protection Agency). OCSPP 850.4150: Vegetative Vigor. In: Office of Chemical Safety and Pollution Prevention, editor. Ecological effects test guidelines. Washington, DC: US Environmental Protection Agency; 2012.

SUPPORTING INFORMATION CAPTIONS

S1 File. Long-term validation of IBC-grass

AUTHOR CONTRIBUTION

Conceptualization: all authors

Formal Analysis: JR

Methodology: JR

Project Administration: SH, FJ, TP

Resources: CM, SH, SM

Software: JR

Supervision: SH, FJ, TP

Validation: all authors

Visualization: JR

Writing – Original Draft Preparation: JR

Writing – Review & Editing: all authors

Figure 1

Click here to download Figure Fig1.tif

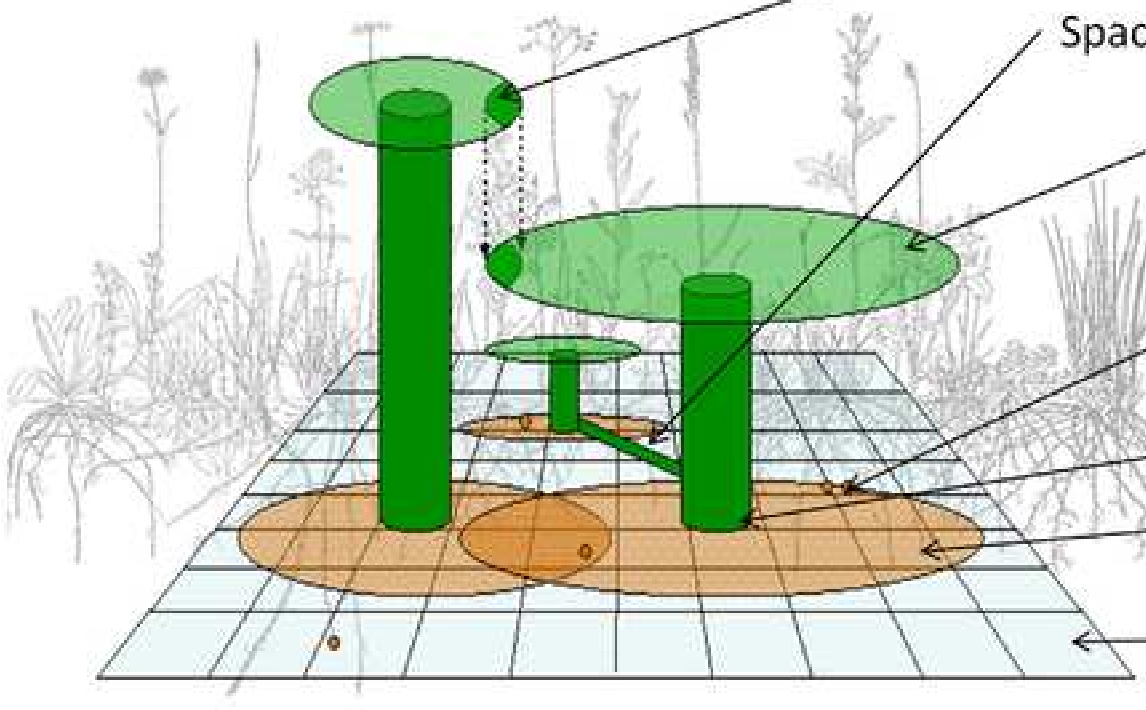

Figure 2

Click here to download Figure Fig2.tif

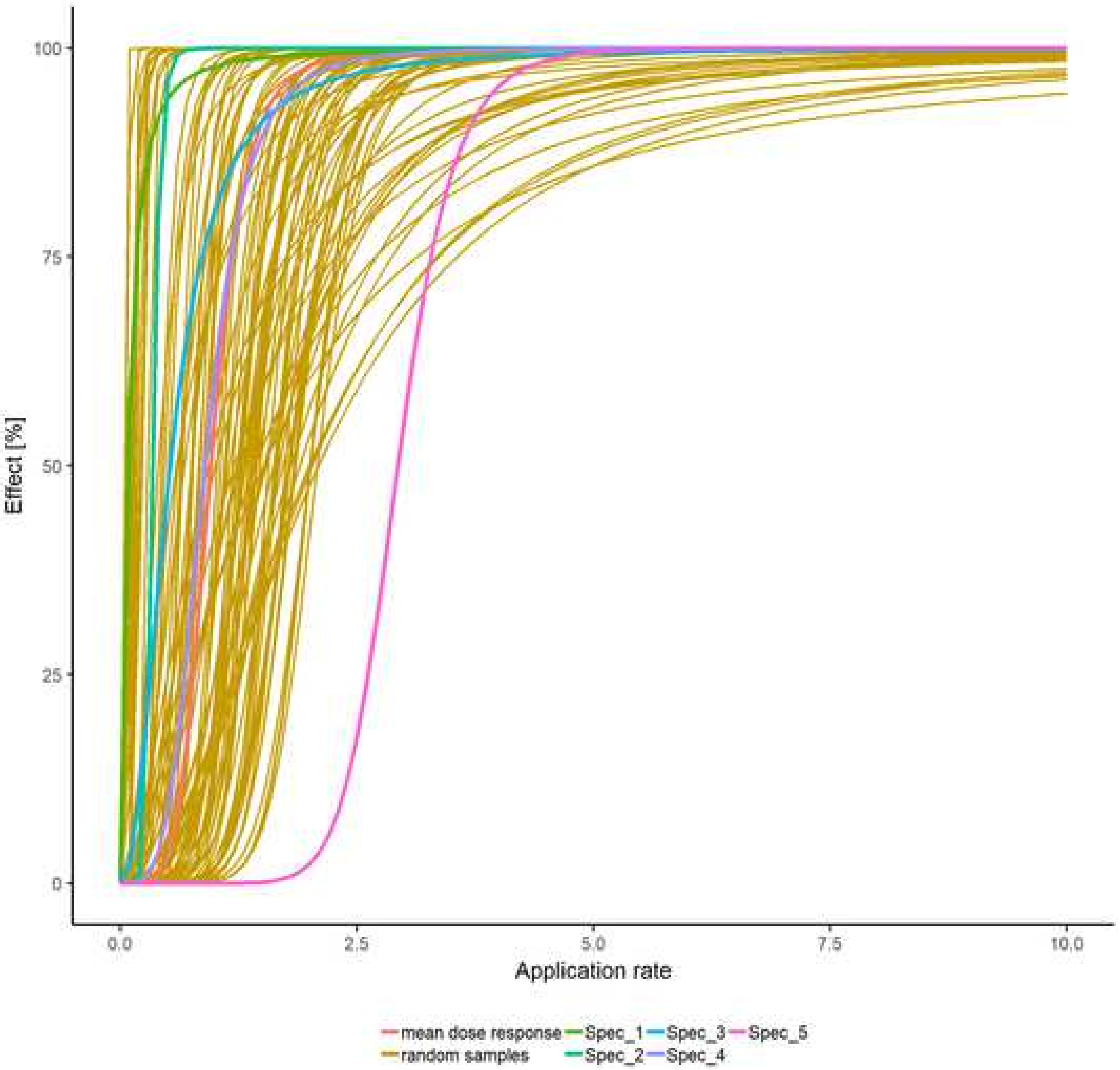

Figure 3

Click here to download Figure Fig3.tif

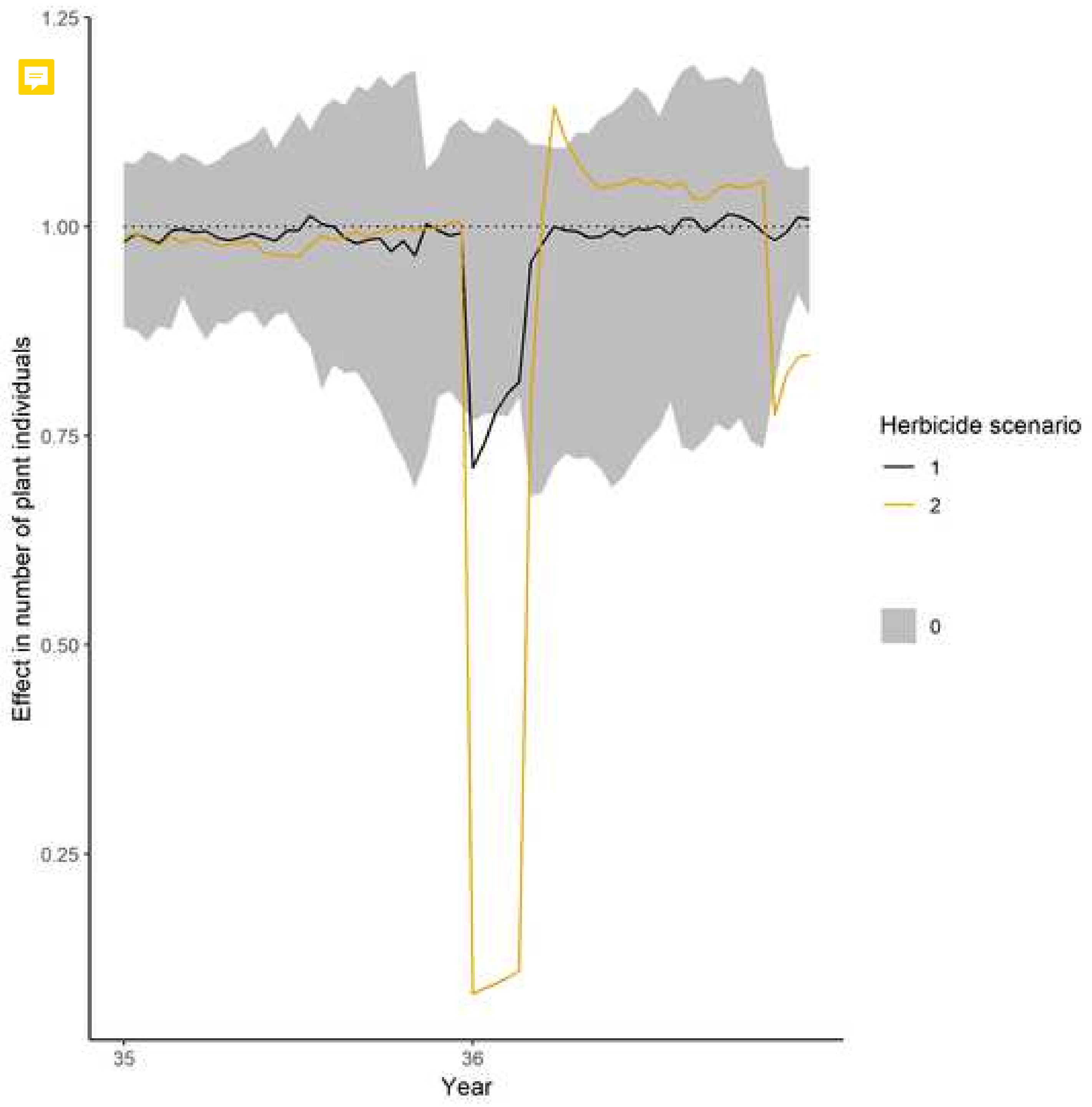

Figure 4

Click here to download Figure Fig4.tif

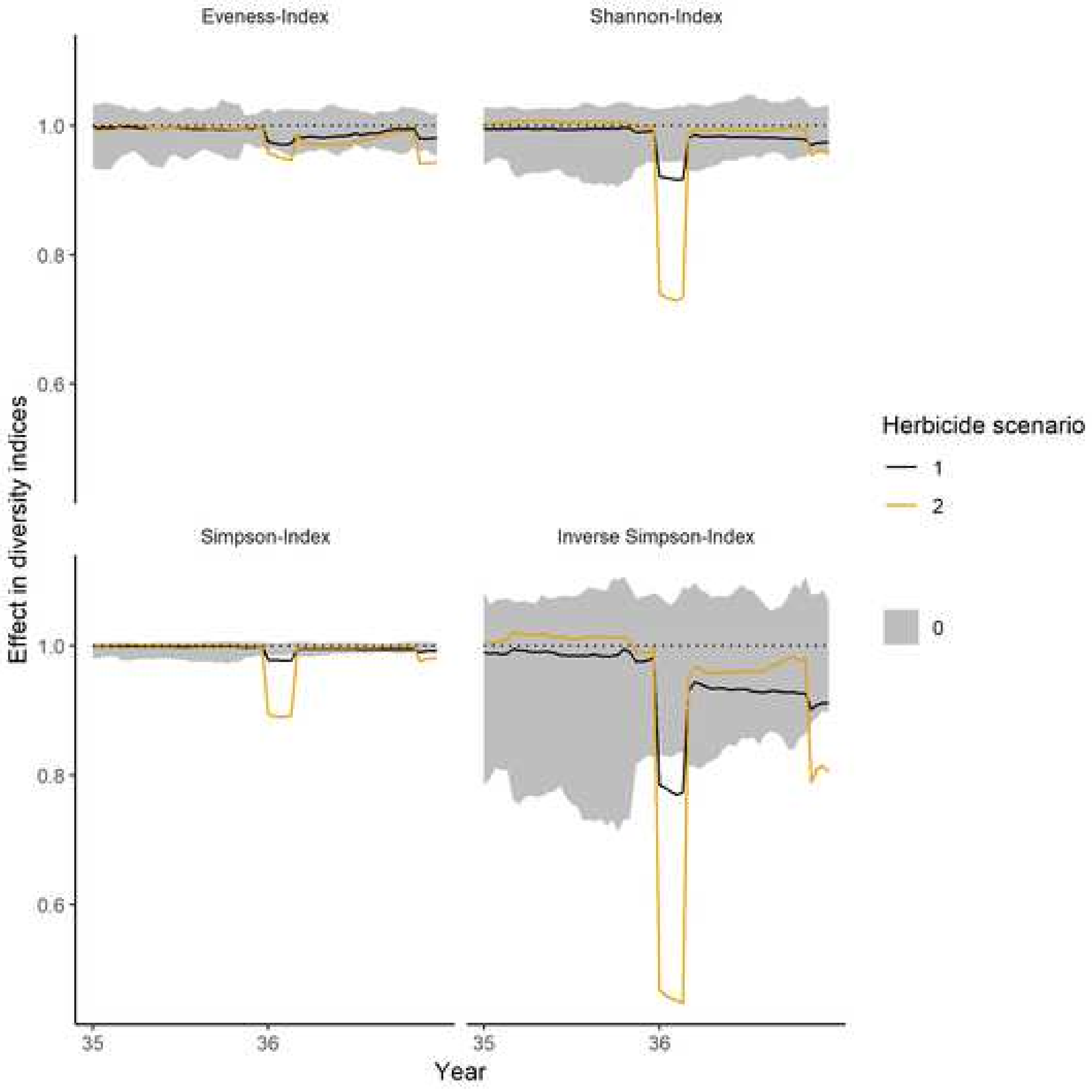

Figure 5

Click here to download Figure Fig5.tif

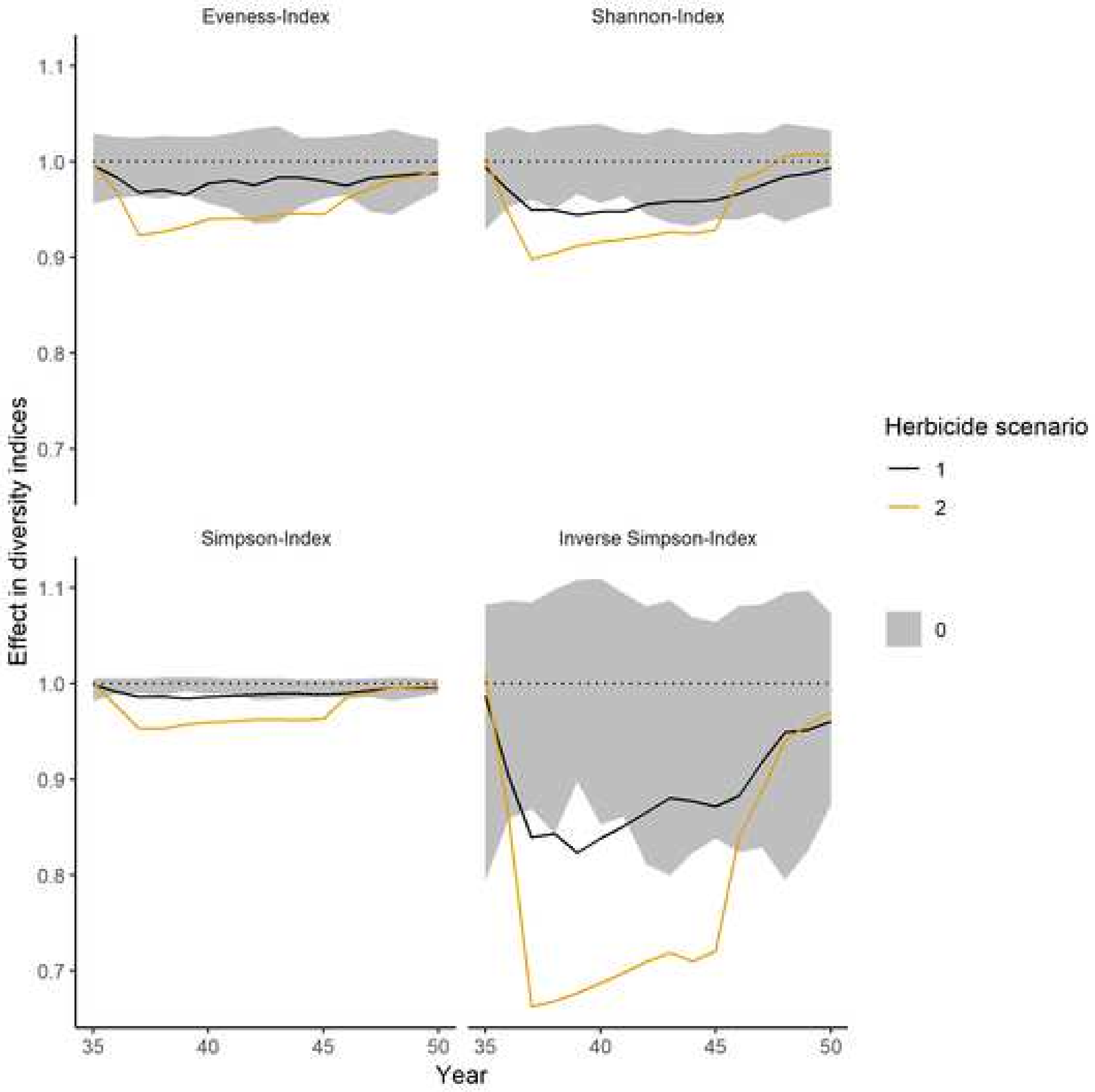

Figure 6 Click here to download Figure Fig6.tif

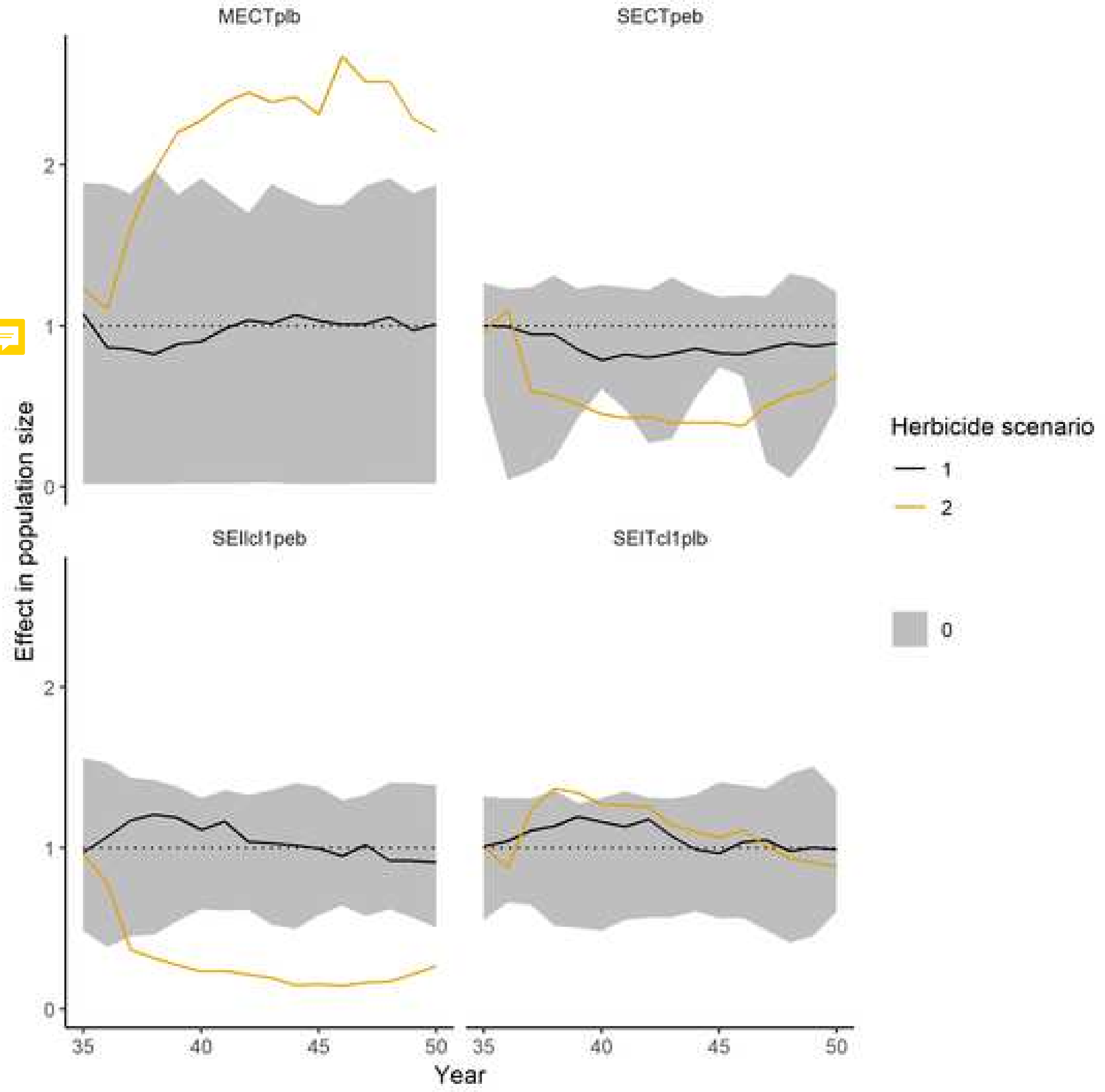

Click here to access/download
Supporting Information
S1 file.docx

Click here to access/download
Data Review URL
https://github.com/JetteReeg/IBCgrassGUI
