# Supplementary material for: Herbicide risk assessments of non-target terrestrial plant communities: A graphical user interface for the plant community model IBC-grass
Source: PLoS One. 2020 Mar 13;15(3):e0230012. doi: 10.1371/journal.pone.0230012 (PMC7069634; doi:10.1371/journal.pone.0230012)
Supplement: Attachment — Submitted filename: Response to Reviewers 2.docx [file pone.0230012.s005.docx]

# Detailed response to reviewers

We would like to thank both reviewers for their comments. As the second reviewer #2 had no further comments, we only response to the comments raised by the reviewer #1.

## Reviewer #1

L 78: I suppose this is the objective. Please, explicit it: "The objective of this paper is..."

We changed the sentence accordingly. (L 78)

Figure 1, L 86ff: Orange and blue colours are not seen in Fig 1

We corrected the figure caption (L86 ff): “Grey boxes indicate processes occurring in each simulated week, green boxes indicate processes occurring only in specific weeks and blue boxes indicate potential herbicide-induced effects that the user can turn on or off. Striped boxes indicate processes the user can adjust and change.”

Table 2 (L 103): The original comment on Table 2 was not answered: "this is not in Table 1. I guess, perennial: cycle more than 2 years long; annual: cycle 1 year long. No biennials?"

We have included the trait in Table 1. Annuals are indeed defined as plants living for 1 year; perennials are defined as plants living for 100 years. Thus, biennials are not included.
